# Integrated analysis of programmed cell death ligand 1 expression reveals increased levels in high-grade glioma

**DOI:** 10.1007/s00432-021-03656-w

**Published:** 2021-05-08

**Authors:** Dorothee Hölzl, Georg Hutarew, Barbara Zellinger, Hans U. Schlicker, Christoph Schwartz, Peter A. Winkler, Karl Sotlar, Theo F. J. Kraus

**Affiliations:** 1 Institute of Pathology, University Hospital Salzburg, Paracelsus Medical University, Müllner Hauptstr. 48, 5020 Salzburg, Austria; 2 Department of Neurosurgery, University Hospital Salzburg, Paracelsus Medical University, Ignatz-Harrer-Str. 79, 5020 Salzburg, Austria

**Keywords:** Glioma, Glioblastoma, Programmed cell death ligand 1, PD-L1, Molecularly targeted therapy

## Abstract

**Purpose:**

Gliomas are the most frequent primary brain tumors of adults. Despite intensive research, there are still no targeted therapies available. Here, we performed an integrated analysis of glioma and programmed cell death ligand 1 (PD-L1) in 90 samples including 58 glioma and 32 control brain tissues.

**Methods:**

To identify PD-L1 expression in glioma, we performed immunohistochemical analysis of PD-L1 tumor proportion score (TPS) using the clinically valid PD-L1 22C3 antibody on 90 samples including controls and WHO grade I–IV gliomas.

**Results:**

We found that PD-L1 is highly expressed in a subfraction of glioma cells. Analysis of PD-L1 levels in different glioma subtypes revealed a strong intertumoral variation of PD-L1 protein. Furthermore, we correlated PD-L1 expression with molecular glioma hallmarks such as MGMT-promoter methylation, *IDH1/2* mutations, *TERT* promoter mutations and LOH1p/19q.

**Conclusion:**

In summary, we found that PD-L1 is highly expressed in a subfraction of glioma, indicating PD-L1 as a potential new marker in glioma assessment opening up novel therapeutic approaches.

## Introduction

Gliomas represent the most frequent primary brain tumors of adults (Louis et al. 2016a). According to the guidelines of the World Health Organization (WHO) for classification of brain tumors, gliomas are assigned to WHO-Grade I–IV tumors representing the degree of aggressiveness (Louis et al. 2016a).

While WHO-Grade I pilocytic astrocytomas (PA) are slow growing gliomas with a good prognosis, WHO-Grade IV glioblastomas (GBM) are highly malignant and diffusely infiltrating brain tumors with a very unfavorable outcome (Louis et al. 2016a). With a reported annual incidence of 3–4 cases per 100,000 population in the western world, GBMs are also the most frequently diagnosed brain tumors in adult patients (Louis et al. 2016a). The highly aggressive clinical behavior of GBMs is also reflected by the histological appearance: They show a high mitotic count, microvascular proliferation and necrosis (Louis et al. 2016a).

For advanced glioma stratification, the 2016 WHO Classification for Central Nervous System (CNS) Tumors integrated molecular genetic findings for advanced tumor classification: (Louis et al. 2016a). Key findings are mutations of the *IDH1 and IDH2* (*Isocitrate Dehydrogenase*), *H3F3A (Histone H3 Family 3A*), *HIST1H3B* and *HIST1H3C* genes, *TERT* (*Telomerase Reverse Transcriptase*) promotor mutations as well as combined chromosomal losses of chromosome 1p and 19q (loss of heterozygosity, LOH) (Louis et al. 2016a). Integrating these molecular findings with histology, there is a severe advance in the prediction of patient outcome (Louis et al. 2007, 2016a, b).

With regard to therapeutic targets, the analysis of the O6-methylguanin–DNA–methyltransferase (MGMT) promotor is of crucial importance (Hegi et al. 2008, 2009; Kaina et al. 2007). The MGMT protein is associated with DNA repair mechanisms, and epigenetic silencing of MGMT transcription by promoter hypermethylation compromises DNA repair mechanisms. Thus, a hypermethylated tumor promoter status has been found to associate with significantly improved survival in patients receiving combined and adjuvant radio-chemotherapy with temozolomide according to the EORTC/NCIC protocol (Hegi et al. 2005). Despite intensive research there is still no targeted therapy available and even by applying temozolomide, the patient outcome is still very unfavorable (Hegi et al. 2004, 2005, 2008, 2012; Hau et al. 2007).

Programmed Cell Death Ligand 1 (PD-L1) is a key player in triggering immune response in human cancers (Campesato et al. 2015; Gatalica et al. 2014; Ohaegbulam et al. 2015). Thereby, PD-L1 interacts with PD-1 (Programmed Cell Death 1) and inhibits immune response by induction of IL-10 (Interleukin) in monocytes (Said et al. 2010). In many tumors, there is an overexpression of PD-L1 that represents a druggable target (Sun et al. 2018; Honda et al. 2017; Kataoka and Ogawa 2016; Kataoka et al. 2016; Isaacsson Velho and Antonarakis 2018; Fan et al. 2019). In lung, breast, gastrointestinal and many other cancers with PD-L1 overexpression showed good response with PD-L1 inhibitors (Reck et al. 2016; Li et al. 2016; Fujita et al. 2015).

However, there is no reliable data available on PD-L1 in glioma with regard to morphological subtypes and genetic profiles. Here, we analyzed PD-L1 expression in 90 different tissue specimens. Thereby, we included 58 glioma samples of WHO Grades I–IV and 32 control brain tissue specimens (16 frontal cortex and 16 frontal white matter samples). Furthermore, we performed integrated analysis of PD-L1 expression and molecular hallmarks of analyzed gliomas.

## Materials and methods

### Tissue collection

We analyzed 90 anonymized tissue samples including 58 glioma and 32 control brains samples. Gliomas were allocated to WHO Grades I to IV and an integrated molecular profiling was performed according to the 2016 WHO classification of CNS tumors (Louis et al. 2016a). All samples were formalin-fixed and paraffin-embedded (FFPE) and stored in the tissue collection of the University Institute of Pathology of the University Hospital Salzburg. Control samples included 16 frontal cortex and 16 frontal white matter samples of post-mortem brains that were formalin-fixed and paraffin-embedded and stored in the tissue collection of the University Institute of Pathology of the University Hospital Salzburg. Details on glioma and control samples including PD-L1 status can be found in Tables 1 and 2.

**Table 1:** Details on glioma samples

ID	Age [years]	Sex	Diagnosis	WHO Grade	IDH1	IDH2	LOH 1p/19q	TERT	MGMT	H3F3A	PD-L1 22C3 positive [%]
T01	39	f	Pilocytic Astrocytoma	I	wt	wt	n.a	wt	Unmethylated	n.a	0
T02	16	m	Pilocytic Astrocytoma	I	wt	wt	n.a	wt	n.a	n.a	0
T03	39	m	Diffuse Astrocytoma	II	p.R132H	wt	wt	wt	Methylated	n.a	0
T04	47	m	Diffuse Astrocytoma	II	p.R132S	wt	wt	wt	Methylated	n.a	0
T05	76	m	Diffuse Astrocytoma	II	wt	wt	wt	C228T	Unmethylated	n.a	0
T06	39	m	Diffuse Astrocytoma	II	p.R132H	wt	wt	wt	Methylated	n.a	1
T07	63	f	Oligodendroglioma	II	p.R132H	wt	1p/19q	C250T	Methylated	n.a	0
T08	54	m	Oligodendroglioma	II	p.R132H	wt	1p/19q	C250T	Methylated	n.a	0
T09	42	m	Oligodendroglioma	II	p.R132H	n.a	1p/19q	n.a	n.a	n.a	0
T10	37	f	Anaplastic Astrocytoma	III	p.R132H	wt	wt	wt	Methylated	n.a	1
T11	67	m	Anaplastic Astrocytoma	III	wt	wt	wt	C250T	Methylated	n.a	7
T12	46	f	Anaplastic Oligodendroglioma	III	p.R132H	wt	1p/19q	wt	Methylated	n.a	45
T13	32	m	Glioblastoma	IV	wt	wt	1p	C228T	Unmethylated	n.a	88
T14	72	m	Glioblastoma	IV	wt	wt	wt	C228T	Methylated	n.a	0
T15	65	m	Glioblastoma	IV	wt	wt	wt	C250T	Methylated	n.a	5
T16	77	f	Glioblastoma	IV	wt	wt	wt	C228T	Methylated	n.a	95
T17	79	f	Glioblastoma	IV	wt	wt	wt	C228T	Unmethylated	n.a	0
T18	28	m	Glioblastoma	IV	wt	wt	wt	wt	Unmethylated	n.a	40
T19	52	f	Glioblastoma	IV	wt	wt	wt	C250T	Unmethylated	n.a	100
T20	44	m	Glioblastoma	IV	wt	wt	n.a	C250T	n.a	n.a	2
T21	78	f	Glioblastoma	IV	wt	wt	n.a	C228T	n.a	n.a	34
T22	45	f	Glioblastoma	IV	wt	wt	n.a	C228T	Methylated	wt	70
T23	77	f	Glioblastoma	IV	wt	wt	n.a	C228T	n.a	n.a	90
T24	50	m	Glioblastoma	IV	wt	wt	n.a	C250T	Methylated	n.a	0
T25	61	m	Glioblastoma	IV	wt	wt	n.a	wt	Unmethylated	n.a	5
T26	70	f	Glioblastoma	IV	wt	wt	n.a	C228T	Unmethylated	n.a	0
T27	69	m	Glioblastoma	IV	wt	wt	n.a	C250T	Unmethylated	n.a	1
T28	83	m	Glioblastoma	IV	wt	wt	n.a	C228T	Unmethylated	n.a	0
T29	51	m	Glioblastoma	IV	wt	wt	n.a	C250T	Methylated	n.a	0
T30	63	f	Glioblastoma	IV	wt	wt	n.a	C250T	Methylated	n.a	0
T31	66	f	Glioblastoma	IV	wt	wt	n.a	C228T	Methylated	n.a	100
T32	77	m	Glioblastoma	IV	wt	wt	n.a	C228T	Unmethylated	n.a	0
T33	58	m	Glioblastoma	IV	wt	wt	n.a	C228T	Unmethylated	wt	0
T34	56	f	Glioblastoma	IV	wt	wt	n.a	C228T	n.a	wt	0
T35	76	f	Glioblastoma	IV	wt	wt	n.a	wt	methylated	n.a	5
T36	72	f	Glioblastoma	IV	wt	wt	n.a	C250T	n.a	n.a	7
T37	25	m	Glioblastoma	IV	wt	wt	n.a	wt	Unmethylated	wt	32
T38	52	m	Glioblastoma	IV	wt	wt	19q	C228T	Methylated	n.a	76
T39	53	m	Glioblastoma	IV	wt	wt	wt	C250T	Unmethylated	n.a	90
T40	79	f	Glioblastoma	IV	wt	wt	wt	wt	Methylated	n.a	55
T41	53	m	Glioblastoma	IV	wt	wt	wt	C228T	Unmethylated	n.a	15
T42	56	m	Glioblastoma	IV	wt	wt	wt	wt	Methylated	n.a	100
T43	60	m	Glioblastoma	IV	wt	wt	n.a	C250T	Methylated	n.a	42
T44	64	m	Glioblastoma	IV	wt	wt	wt	C228T	Methylated	n.a	55
T45	66	m	Glioblastoma	IV	wt	wt	n.a	C228T	Methylated	n.a	98
T46	82	f	Glioblastoma	IV	wt	wt	n.a	C228T	Unmethylated	n.a	0
T47	72	m	Glioblastoma	IV	wt	wt	n.a	C228T	Unmethylated	n.a	1
T48	87	m	Glioblastoma	IV	wt	wt	n.a	C228T	Unmethylated	n.a	28
T49	56	m	Glioblastoma	IV	wt	wt	19q	C228T	Methylated	n.a	0
T50	64	m	Glioblastoma	IV	wt	wt	1p	C250T	n.a	n.a	48
T51	59	m	Glioblastoma	IV	wt	wt	n.a	C250T	n.a	n.a	90
T52	71	m	Glioblastoma	IV	wt	wt	wt	wt	Unmethylated	n.a	10
T53	50	f	Glioblastoma	IV	wt	wt	wt	C228T	Unmethylated	n.a	0
T54	58	m	Glioblastoma	IV	wt	wt	1p	C250T	Methylated	n.a	24
T55	75	f	Glioblastoma	IV	wt	wt	1p	C250T	Unmethylated	n.a	90
T56	41	m	Glioblastoma	IV	wt	wt	wt	C250T	Unmethylated	n.a	86
T57	39	m	Diffuse Midline Glioma	IV	wt	wt	n.a	C228T	Unmethylated	K27M	2
T58	33	f	Diffuse Midline Glioma	IV	wt	wt	n.a	wt	Methylated	K27M	0

Indicated are details on all 58 glioma samples

*wt* wild type, *n.a.* not available

**Table 2:** Details on control samples

ID	Age [years]	Sex	Region	PD-L1 22C3 positive [%]
C01	95	m	Frontal Cortex	0
C02	56	m	Frontal Cortex	0
C03	62	m	Frontal Cortex	0
C04	65	f	Frontal Cortex	0
C05	92	f	Frontal Cortex	0
C06	75	f	Frontal Cortex	0
C07	75	f	Frontal Cortex	0
C08	87	f	Frontal Cortex	0
C09	54	f	Frontal Cortex	0
C10	67	f	Frontal Cortex	0
C11	79	f	Frontal Cortex	0
C12	69	f	Frontal Cortex	0
C13	89	f	Frontal Cortex	0
C14	52	m	Frontal Cortex	0
C15	59	m	Frontal Cortex	0
C16	54	m	Frontal Cortex	0
W01	95	m	Frontal White Matter	0
W02	56	m	Frontal White Matter	1
W03	62	m	Frontal White Matter	0
W04	65	f	Frontal White Matter	0
W05	92	f	Frontal White Matter	0
W06	75	f	Frontal White Matter	0
W07	75	f	Frontal White Matter	0
W08	87	f	Frontal White Matter	0
W09	54	f	Frontal White Matter	0
W10	67	f	Frontal White Matter	0
W11	79	f	Frontal White Matter	0
W12	69	f	Frontal White Matter	0
W13	89	f	Frontal White Matter	0
W14	52	m	Frontal White Matter	0
W15	59	m	Frontal White Matter	0
W16	54	m	Frontal White Matter	1

Indicated are details on all 32 control samples

### Molecular genetic characterization of gliomas

Molecular genetic analysis of glioma samples was performed as previously described (Kraus et al. 2020). In brief, DNA extraction for molecular pathological analysis was performed of microscopically identified representative tumor tissues with at least 90% of viable tumor cells applying the Maxwell system (Promega) according to the manufacturer’s instructions. *IDH1* and *IDH2* and *BRAF* hot spot mutations were analyzed applying the AmpliSeq for Illumina Cancer Hotspot Panel v2 (Illumina) on an Illumina MiniSeq next generation sequencing device (Illumina) according to the manufacturer’s protocols. Hot spot loci of TERT promoter, *H3F3A*, *HIST1H3B* and *HIST1H3C* genes were analyzed by Sanger sequencing as described previously (Kraus et al. 2020). MGMT promotor methylation was assessed by methylation specific PCR (MSP) and bisulfite sequencing (Kraus et al. 2015a, b). Assessment of 1p/19q status was performed by Fluorescence in situ hybridization (FISH) applying ZytoLight 1p/1q and 19q/19p probe sets (ZytoVision) following the manufacturer’s protocols. According to the guidelines of the current WHO classification, 1p/19q status was assessed in all *IDH* mutated glioma, since loss of 1p and 19q is only occurring in gliomas harboring *IDH* mutations (Louis et al. 2016a).

### Immunohistochemical analysis

Routine immunohistochemistry performed on glioma samples included antibodies against GFAP, Ki67 and PHH3. PD-L1 expression was assessed applying the PD-L1 22C3 antibody (M3653 antibody kit, Dabo). Quantification of PD-L1 levels were performed by DH, TFJK and GH using the tumor proportion score (TPS) (Li et al. 2017; Neuman et al. 2016; Roge et al. 2017). All immunohistochemical stains were performed on a Ventana BenchMark Ultra device (Roche) according to the manufacturer’s protocols.

### Computational data analysis

Statistical analysis was performed using Prism 9 (GraphPad) software suite. As statistical tests, we applied *t* test and one-way ANOVA with uncorrected Fisher’s Test. Statistical significance was assumed for *p* values < 0.05.

## Results

### PD-L1 is expressed in human gliomas

**Fig. 1:**
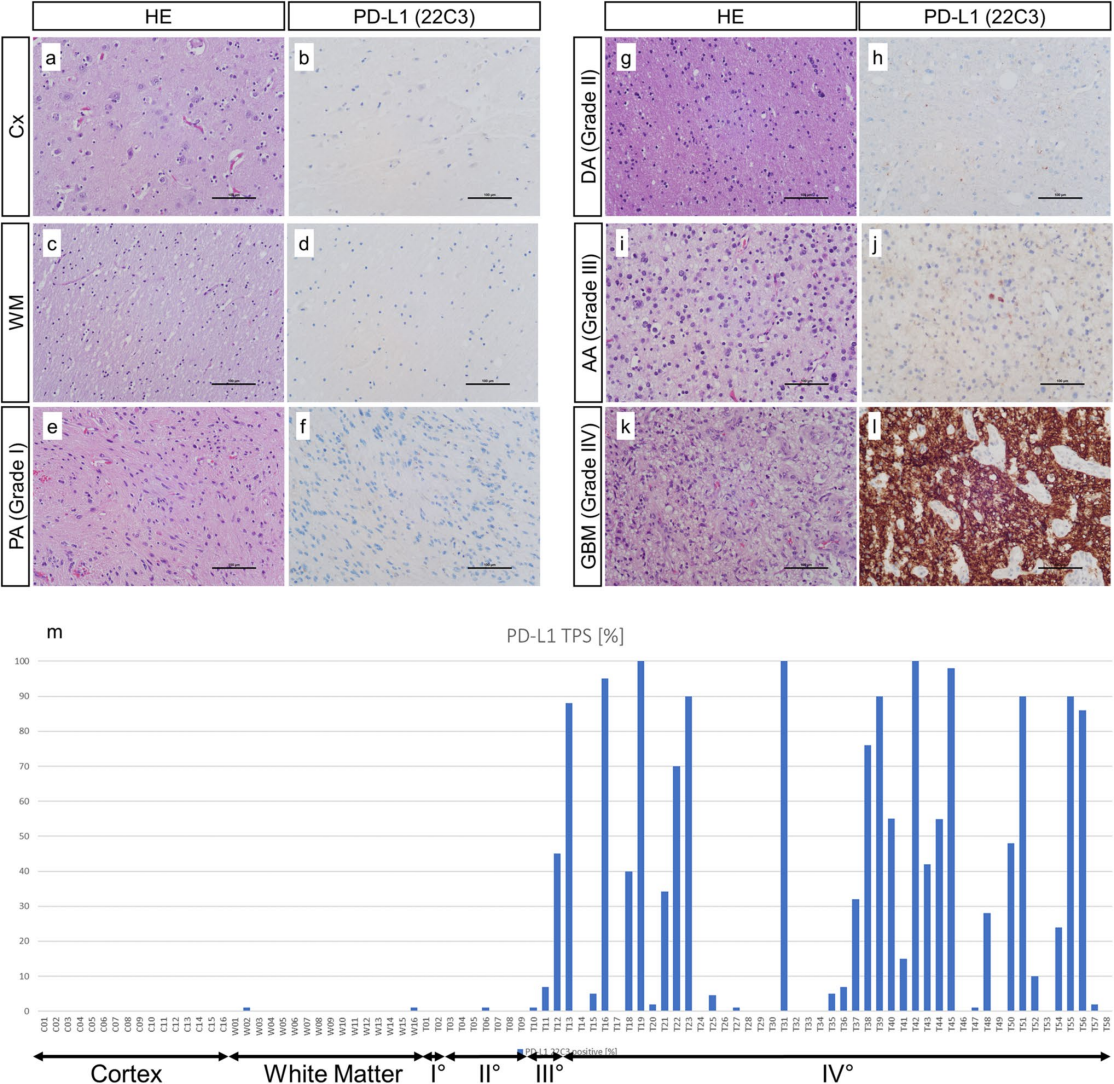
PD-L1 expression in healthy brain tissue and glioma. Analysis of 90 tissue samples showed no PD-L1 expression in healthy cortex (**a**, **b**) and white matter regions (**c**, **d**). There was no noteworthy PD-L1 expression in low grade glioma, i.e., WHO grade I pilocytic astrocytoma (**e**, **f**) and diffuse astrocytoma (**g**, **h**). In high grade glioma there was an uneven PD-L1 expression with strong intertumoral heterogeneity in WHO grade III anaplastic astrocytoma (**i**,** j**) and glioblastoma (**k**, **l**). Distinct PD-L1 TPS scores of all 90 analyzed samples is presented in m. *CX* cortex, *WM* white matter, *PA* pilocytic astrocytoma, *DA* diffuse astrocytoma, *AA* anaplastic astrocytoma, *GBM* glioblastoma. **a**–**l** Scale bar: 100 µm

To evaluate the significance of PD-L1 expression in gliomas, we used the PD-L1 22C3 antibody and performed immunohistochemical analysis in 90 tissue samples. These samples include 58 gliomas of WHO grades I, II, III and IV and 32 control brain samples including cortex and white matter regions. We found that there was no PD-L1 expression in control tissue, i.e., cortex (*n* = 16, Fig. 1a, b) and white matter (*n* = 16, Fig. 1c, d). In gliomas, we found uneven PD-L1 expression. Low grade gliomas consisting of WHO grade I pilocytic astrocytomas (*n* = 2, Fig. 1e, f) and WHO grade II diffuse gliomas (*n* = 7, Fig. 1g, h) did not show noteworthy PD-L1 expression. High grade gliomas consisting of WHO grade III anaplastic gliomas (*n* = 3, Fig. 1i, j) and WHO grade IV glioma (*n* = 46, Fig. 1k, l), showed intermediate to high PD-L1 expression. PD-L1 tumor proportion scores (TPS) of all 90 analyzed samples can be found in Fig. 1m.

### PD-L1 is significantly overexpressed in high grade gliomas

**Fig. 2:**
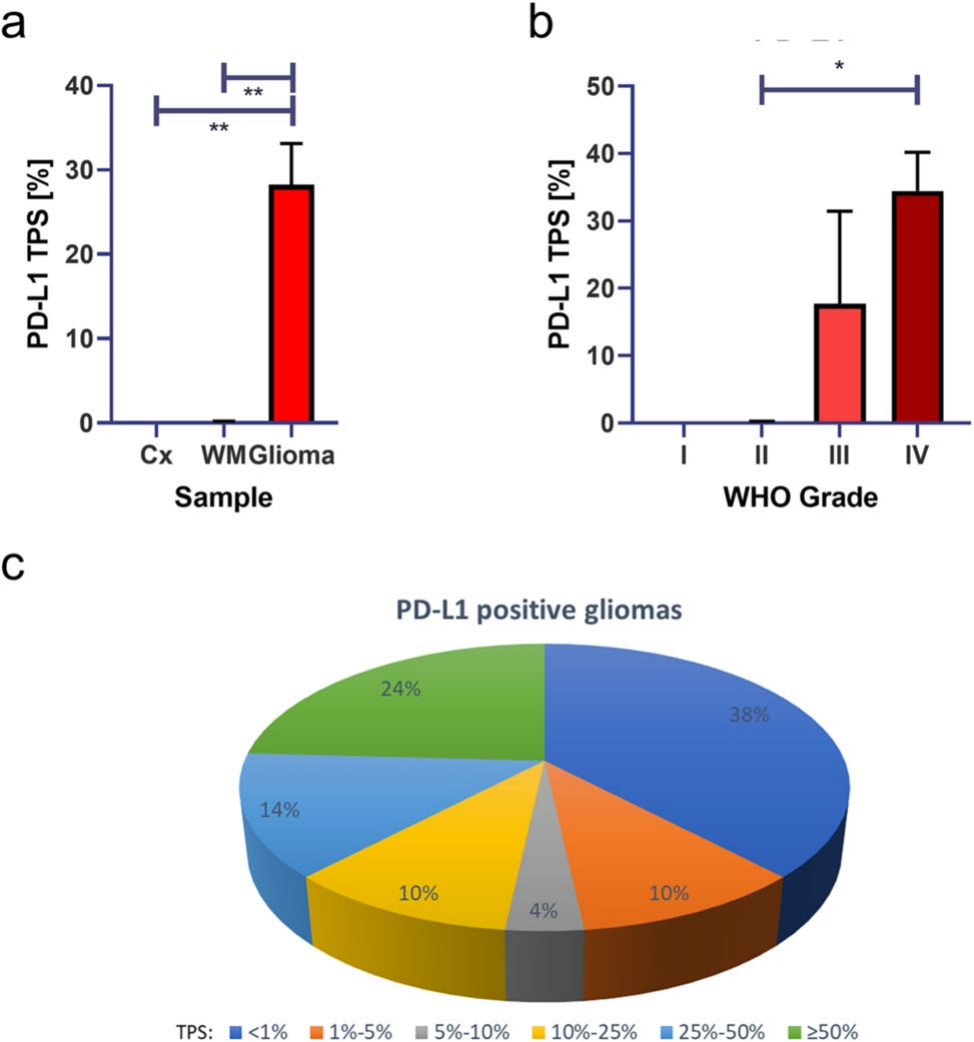
Statistical analysis of PD-L1 expression. Statistical analysis of PD-L1 expression showed significant overexpression of PD-L1 in glioma compared with healthy cortex (**a**) and white matter (**b**). Analysis of WHO grade I, II, III and IV glioma showed significant overexpression in high grade glioblastoma compared with low grade diffuse glioma. Analysis of individual TPS showed PD-L1 expression in glioma showed TPS of ≥ 50% in 27% of gliomas, TPS of 25–50% in 12% of gliomas, TPS of 10–25% in 10% of gliomas, TPS of 5–10% in 2% of gliomas, TPS of 1–5% in 9% of gliomas, and TPS of < 1% in 40% of gliomas (**c**). **a**, **b** Indicated are mean and SEM. *p < 0.05, **p < 0.01

A detailed analysis of PD-L1 expression in all 90 tissues specimen revealed significant overexpression of PD-L1 in glioma compared with healthy brain tissue: There was a statistically significant overexpression in glioma compared to cortex (*p* < 0.01, Fig. 2a) and white matter (*p* < 0.01, Fig. 2a). Analyzing PD-L1 expression and WHO grade confirmed high PD-L1 expression in high grade gliomas with a significant overexpression in WHO grade IV glioblastomas (*p* < 0.05, Fig. 2b). A detailed analysis of PD-L1 expression in glioma showed that 24% of all glioma showed TPS of ≥ 50%, 14% showed TPS of 25–50%, 10% showed TPS of 10–25%, 4% showed TPS of 5–10%, 10% showed TPS of 1–5% and 38% showed TPS of < 1% (Fig. 2c).

### Integrated analysis of PD-L1 expression and molecular glioma hallmarks

**Fig. 3:**
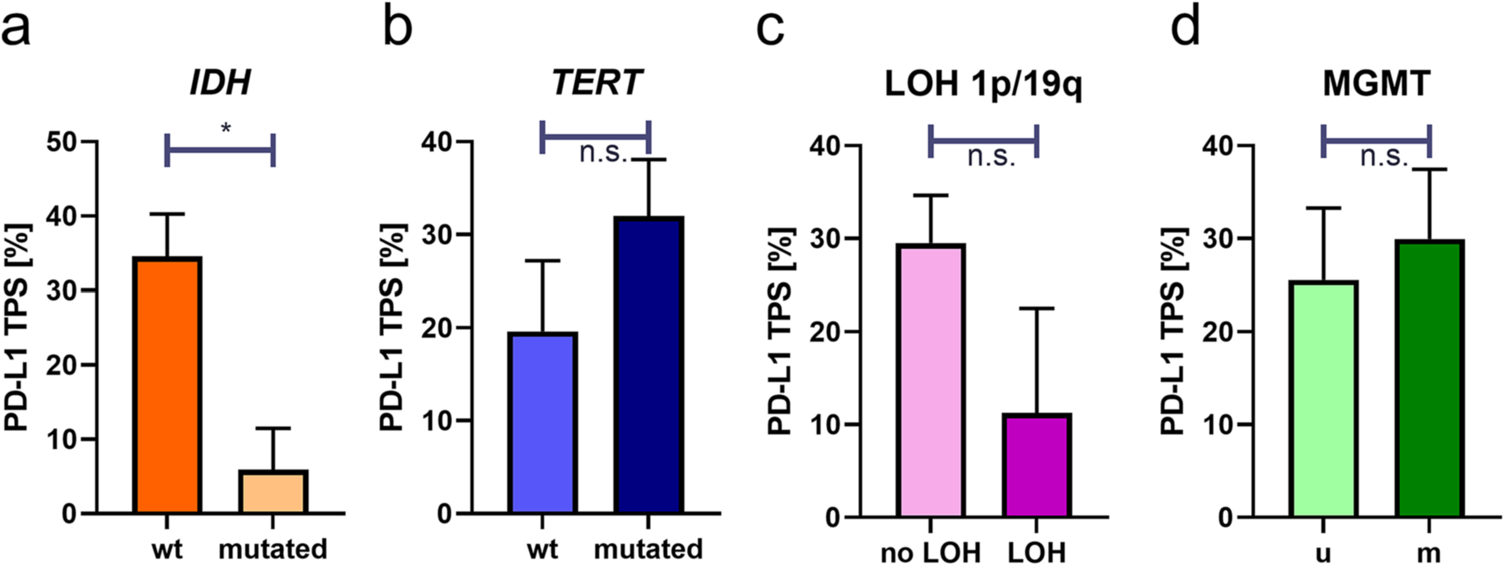
Integrated analysis of PD-L1 expression and molecular genetic hallmarks of glioma. Analysis of PD-L1 expression and IDH status showed higher expression of PD-L1 in IDH wild type compared to IDH R132H mutated glioma (**a**). In case of TERT promoter mutation there was higher PD-L1 expression in TERT C228T and C250T mutated glioma (**b**). Analysis of LOH 1p/19q showed higher expression of PD-L1 in gliomas without LOH 1p/19q (**d**). Analysis of MGMT promoter methylation showed higher expression of PD-L1 in methylated glioma (**c**). Indicated are mean and SEM. *m* methylated, *u* unmethylated; *n.s.* not significant (*p* > 0.05), *p < 0.05

Since gliomas show distinct molecular hallmarks, we next performed an integrated analysis of PD-L1 TPS and molecular genetic status: *IDH* mutation, *TERT* promoter mutation, MGMT promoter methylation and loss of heterozygosity of 1p and 19q (LOH 1p/19q). Interestingly, *IDH* wild-type glioma (*n* = 46) showed a significant higher expression of PD-L1 compared with *IDH* mutated gliomas (*n* = 8, *p* < 0.05, Fig. 3a). Due to the different biological backgrounds (Louis et al. 2016a) of pilocytic astrocytomas and *H3F3A* mutated diffuse midline gliomas, these samples were excluded from *IDH* analysis. In case of *TERT* promoter mutation, *TERT* mutated gliomas (*n* = 42) showed higher PD-L1 expression compared with *TERT* wild-type gliomas (*n* = 5, *p* > 0.05) (Fig. 3b). An analysis of loss of heterozygosity of 1p and 19q (LOH 1p/19q) showed higher PD-L1 expression in gliomas without LOH 1p/19q (*n* = 54) compared to LOH 1p/19q aberrant gliomas (*n* = 4, *p* > 0.05 (Fig. 3c). Analysis of PD-L1 expression and MGMT promoter methylation showed higher PD-L1 expression in MGMT methylated glioma (*n* = 26) compared to MGMT unmethylated glioma (*n* = 23, *p* > 0.05) (Fig. 3d).

## Discussion

Despite intensive research, there are still no curative therapies available for GBM patients (Louis et al. 2016a). One milestone in glioblastoma therapy was the discovery of the connection between methylation of the MGMT promotor (Hegi et al. 2008, 2009; Kaina et al. 2007) and tumor response to chemotherapy using temozolomide in 2005 (Hegi et al. 2005). However, since then there have not been any significant advances in glioblastoma therapy.

In anti-tumor therapy, PD-L1 is already a key player in personalized medicine, since it represents a druggable target (Sun et al. 2018; Honda et al. 2017; Kataoka and Ogawa 2016; Kataoka et al. 2016; Isaacsson Velho and Antonarakis 2018; Fan et al. 2019). In many tumors, such as lung, breast, gastrointestinal PD-L1 inhibitors show great advances in patient treatment (Reck et al. 2016; Li et al. 2016; Fujita et al. 2015). Thereby, the expression profile of PD-L1 is assessed immunohistochemically.

Here, we assessed PD-L1 expression using the tumor proportion score (TPS), i.e., the percentage of PD-L1 positive tumor cells compared with all vital tumor cells (Li et al. 2017; Neuman et al. 2016; Roge et al. 2017) to assess PD-L1 expression in gliomas, and thus to evaluate the feasibility of PD-L1 inhibitors in highly aggressive brain tumors.

Our analysis of PD-L1 expression revealed that there are high PD-L1 expression levels in high grade glioma with a high interindividual variation (Fig. 1). While control cortex and white matter tissues showed mean PD-L1 TPS of 0%, gliomas showed significantly increased PD-L1 TPS with a mean of 28% in all 58 gliomas (Fig. 2a). A further subgroup analysis of different WHO grades showed that PD-L1 expression can be found predominantly in high grade gliomas with mean amounts of 18% positive tumor cells in WHO grade III gliomas and 34% positive tumor cell in WHO grade IV glioblastomas, respective (Fig. 2b). Furthermore, we performed integrated analysis of molecular key hallmarks in glioma (*IDH*, *TERT*, MGMT methylation) and PD-L1 expression. Interestingly, we found significantly higher PD-L1 expression in *IDH* wild-type glioma (mean amounts of 32%) compared with IDH mutated gliomas (mean amounts of 6%, *p* < 0.05, Fig. 3a). In terms of *TERT*, we found higher PD-L1 expression in TERT mutated glioma (mean amounts of 32%) compared with *TERT* wild-type glioma (mean amounts of 20%, *p* > 0.05, Fig. 3b). An analysis of loss of heterozygosity of 1p and 19q (LOH 1p/19q) showed higher PD-L1 expression in gliomas without LOH 1p/19q (mean amounts of 30%) compared with LOH 1p/19q aberrant gliomas (mean amounts of 11%, *p* > 0.05, Fig. 3c). Analysis of MGMT promoter methylation revealed higher PD-L1 expression in MGMT methylated glioma (mean amounts of 30%) compared with MGMT unmethylated glioma (mean amounts of 26%, Fig. 3d).

Considering the gliomagenesis and aggressiveness of glioma, Louis et al. (2016a) these findings are of high therapeutic impact: while *IDH* mutation is a key pathway in gliomagenesis of WHO grade II and III gliomas and secondarily progressed WHO grade IV glioblastomas, *IDH* wild-type is a typical hallmark of primary WHO grade IV glioblastomas. Thus, the finding of high PD-L1 expression in *IDH* wild-type primary glioblastomas is of severe clinical importance opening new therapeutic approaches in therapy of highly aggressive glioblastoma. Vice versa to *IDH* mutations, TERT mutations are predominantly present in glioblastoma. Thus, the result of high PD-L1 expression in TERT mutated gliomas may also be of high clinical importance for therapy of highly aggressive glioblastomas.

Since the importance of PD-L1 has already been established as personalized medicine target in other tumor entities (Sun et al. 2018; Honda et al. 2017; Kataoka and Ogawa 2016; Kataoka et al. 2016; Isaacsson Velho and Antonarakis 2018; Fan et al. 2019; Reck et al. 2016; Li et al. 2016; Fujita et al. 2015) our findings in glioma may also open new therapeutic approaches in future brain tumor therapy. Thereby, our results are well in line with published data: Nduom et al. found that PD-L1 expression can be found in a subfraction of glioblastoma (Nduom et al. 2016). Thereby high PD-L1 expression is correlated with worse outcome (Nduom et al. 2016). Heiland et al. also report of high PD-L1 expression in glioblastoma with predominance of IDH wild-type glioblastomas (Heiland et al. 2017). Berghoff et al. analyzed PD-L1 expression and tumor infiltrating lymphocytes (TIL) in diffuse glioma and found that high PD-L1 expression and prominent TILs are predominantly present in *IDH* wild-type glioma compared with *IDH* mutant glioma (Berghoff et al. 2017). Hao et al. performed a meta-analysis of PD-L1 expression in glioblastoma and also confirmed that high PD-L1 expression can be found predominantly in glioblastoma with unfavorable outcome (Hao et al. 2020). This finding is well in accordance with our results demonstrating that highly aggressive *IDH* wild-type gliomas show higher PD-L1 expression. In contrast to previous studies, Nduom et al. (2016), Heiland et al. (2017), Hao et al. (2020) we performed PD-L1 expression using the widely accepted and clinically applicable PD-L1 22C3 clone (M3653 antibody kit, Dako) and the tumor proportion score (TPS). Thus, our approach using the PD-L1 22C3 antibody and TPS to evaluate PD-L1 expression opens the way for monoclonal antibody therapies such as prembolizumab in a clinical setting (Ilie et al. 2017). However, the significance of this study is limited due to the low number of cases in distinct subgroups of glioma, such as oligodendroglioma and diffuse midline glioma. Thus, further studies with an increased number of cases will be needed to validate these results. A further limitation of this study is that only a limited set of molecular parameters was assessed, e.g., there was no molecular assessment of the *BRAF* status in pilocytic astrocytomas. Furthermore, there is one case of diffuse astrocytoma with *IDH* wild-type status and *TERT* mutation included in this study. This is a very untypical genotype–phenotype combination and there should be further molecular assessment according to the cIMPACT guidelines (Louis et al. 2020; Gonzalez Castro and Wesseling 2021) including copy number profiling (CNP) to further characterize such cases and to assess, if the underlying biology is that of glioblastoma *IDH* wild type.

In summary, our findings demonstrate the significance of PD-L1 testing in glioma enabling new individualized strategies for molecularly targeted therapy in highly aggressive brain tumors.

## Data Availability

Details on data can be found in Tables 1 and 2, further information on the datasets used and/or analyzed during the current study are available from the corresponding author on reasonable request.
